# Phase II trial of isoflavone in prostate-specific antigen recurrent prostate cancer after previous local therapy

**DOI:** 10.1186/1471-2407-8-132

**Published:** 2008-05-11

**Authors:** John M Pendleton, Winston W Tan, Satoshi Anai, Myron Chang, Wei Hou, Kathleen T Shiverick, Charles J Rosser

**Affiliations:** 1 Division of Urology, The University of Florida, Jacksonville, Florida 32209, USA; 2 Department of Hematology-Oncology, Mayo Clinic, Jacksonville, FL 32224, USA; 3 Department of Epidemiology and Health Policy Research, The University of Florida, Gainesville, Florida 32610, USA; 4 Department of Pharmacology, The University of Florida, Gainesville, Florida 32610, USA

## Abstract

**Background-:**

Data exist that demonstrate isoflavones' potent antiproliferative effects on prostate cancer cells. We evaluated the efficacy of isoflavone in patients with PSA recurrent prostate cancer after prior therapy. We postulated that isoflavone therapy would slow the rate of rise of serum PSA.

**Methods-:**

Twenty patients with rising PSA after prior local therapy were enrolled in this open-labeled, Phase II, nonrandomized trial (Trial registration # NCT00596895). Patients were treated with soy milk containing 47 mg of isoflavonoid per 8 oz serving three times per day for 12 months. Serum PSA, testosterone, lipids, isoflavone levels (genistein, daidzein, and equol), and quality of life (QOL) were measured at various time points from 0 to 12 months. PSA outcome was evaluated.

**Results-:**

Within the mixed regression model, it was estimated that PSA had increased 56% per year before study entry and only increased 20% per year for the 12-month study period (*p *= 0.05). Specifically, the slope of PSA after study entry was significantly lower than that before study entry in 6 patients and the slope of PSA after study entry was significantly higher than before study entry in 2 patients. For the remaining 12 patients, the change in slope was statistically insignificant. Nearly two thirds of the patients were noted to have significant levels of free equol in their serum while on therapy.

**Conclusion-:**

Dietary intervention with isoflavone supplementation may have biologic activity in men with biochemical recurrent prostate cancer as shown by a decline in the slope of PSA. This study may lend support to the literature that nutritional supplements have biologic activity in prostate cancer and therefore, further studies with these agents in randomized clinical trials should be encouraged.

## Background

Environmental factors have been investigated as possible etiologic influences for the development of prostate cancer, including occupational exposures, smoking, alcohol consumption, sexual practices, venereal transmitted diseases, and vasectomy. Only dietary influences have shown any promise[1]. The most provocative data supporting the influence of dietary factors on the clinical incidence of prostate cancer come from international studies and from studies of disease expression in immigrant populations. Historically, the incidence of prostate cancer in Japan has been extremely low. However, as Japanese men migrate to Hawaii or the U.S. mainland and subsequently adopt a western culture, their incidence of prostate cancer rapidly approaches that of Caucasian Americans [3,4]. The diets of native Chinese and Japanese are rich in fiber and low in saturated fat. These cultural differences may contribute to the lower rates of clinical prostate cancer in the Far East compared to Northern Europe and North America. In addition, recent evidence suggests that soy consumption contributes to the lower clinical incidence of prostate cancer in Asian countries [5].

One of the several compounds in soy is isoflavone, a soybean protease inhibitor. *In vitro *experimental data suggest that isoflavones are cancer preventive agents [6]. Genistein glycosides account for more than two-thirds of the total soybean isoflavone content [7]. Genistein is known to inhibit both androgen-dependent and androgen-independent prostate cancer cell growth *in vitro*. It is speculated that the mechanisms by which genistein exerts its effect are related to the inhibition of protein tyrosine kinase, DNA topoisomerases, angiogenesis, as well as its effect on cellular differentiation and apoptosis [8].

Daidzein is another major constituent of total soybean isoflavone content. It is speculated that some humans possess the necessary intestinal flora to convert daidzein to equol, a molecule similar to estrogen [9]. Thus, isoflavones may act through hormonal manipulation. If isoflavones change the hormonal milieu, it is quite possible that their mechanism of action is centered on the androgen receptor (AR). AR activity has been implicated as pivotal in several phases of prostate cancer: origin, transition to androgen independent status, and progression. Linkage studies suggest that the activity of the AR might be correlated with tumor incidence and aggressiveness. Researchers have found that a decreased repeat length of an AR polymorphism correlates with an increased risk of and aggressiveness of prostate cancer [10].

We set out to determine if dietary supplementation is indeed a potential therapeutic option, by evaluating the efficacy of isoflavone in subjects with prostate-specific antigen (PSA) recurrent prostate cancer after radiation therapy or radical prostatectomy. Herein, we report the serum PSA, CAG androgen receptor polymorphism, and quality of life results of our phase II, prospective study.

## Methods

This study was performed after review and approval by the Institutional Review Board of the University of Florida at Jacksonville, an inner-city hospital serving Duval County. The study was an investigator initiated, industry sponsored (Hain-Celestial, Denver CO), phase II, non-randomized clinical trial. The study was registered with National Institute of Health (registration # NCT00596895).

### Study Design

Sample size determination to detect a reduction of 10% in PSA from initial levels [actual calculation as log_10 _(PSA) with a standard deviation of 37% of PSA level] required 27 patients to have a significance of p = 0.05 and a power of 60%. However, due to poor accrual, the study was closed after the enrollment of 20 patients. With the sample size of 20, a reduction of 12% in PSA can be detected with the same significance level and power.

Twenty patients were enrolled in this study from May 2004 to January 2007. Eligibility criteria included: able to give informed consent, adenocarcinoma of the prostate (no small cell component), and prior treatment with radiation (at least 6200 cGy, n = 9) or radical prostatectomy (n = 11) for clinically localized disease (clinical T1 or T2). Table 1 shows the demographic, clinical, and pathologic characteristics of the study cohort. All patients had demonstrated evidence of biochemical failure within a median of 6 ± 3 years after treatment. Biochemical failure was defined as a) detectable and rising PSA after surgery or b) three consecutive PSA rises after radiation therapy [11].

**Table 1:** Demographic, Clinical, and Pathologic Characteristics of 20 men with biochemical recurrent prostate cancer after previous therapy

Patient No.	Age (yr.)	Race	Serum PSA at Initial Diagnosis (ng/ml)	Gleason Score at Initial Diagnosis	Clinical Stage at Initial Diagnosis	Initial Treatment	Length of F/U months
1	62	White	UNK	3+4 = 7	T3aNxMx	RP	12
2	67	White	4.8	3+3 = 6	T2xNxMx	Brachy/EBRT/ADT	12
3	67	White	UNK	3+3 = 6	T2xNxMx	RP	12
4	78	White	4.7	3+4 = 7	T2aNxMx	Brachy/EBRT/ADT	6, started ADT
5	77	African American	14.08	UNK	T3aNxMx	RP/EBRT	12
6	75	White	8.28	4+5 = 9	T2cNxMx	ADT/RP	12
7	69	White	10.36	3+3 = 6	T1cNxMx	EBRT	12
8	71	White	UNK	UNK	UNK	RP/Salvage EBRT	12
9	75	White	UNK	4+4 = 8	T2bNxMx	EBRT/ADT	12
10	77	White	8.1	4+5 = 9	T3bNxMx	RP	3, started ADT
11*	79	White	UNK	2+3 = 5	T1cNxMx	EBRT	9, withdrew due to side effects
12	68	White	UNK	4+4 = 8	T2aNxMx	RP/Adjuvant EBRT	6, started ADT
13	76	African American	11.13	3+3 = 6	T2aNxMx	EBRT	12
14	79	White	4.0	3+4 = 7	T1cNxMx	RP/Adjuvant EBRT	12
15	73	White	10	3+2 = 5	T1cNxMx	EBRT	12
16	60	White	9.2	3+4 = 7	T1cNxMx	RP/Adjuvant EBRT	12
17	63	White	17.3	4+3 = 7	T1cNxMx	EBRT	12
18	70	White	9.8	3+3 = 6	T2xNxMx	RP/salvage EBRT	12
19*	76	African American	4.1	3+4 = 7	T2bNxMx	RP	6, lost to F/U
20	77	White	8.7	UNK	T2aNxMx	EBRT	6, lost to F/U
Median	73	---	9.2	7	---	---	12

*, noncompliant

Brachy, brachytherapy

EBRT, external beam radiation therapy

RP, radical prostatectomy

ADT, androgen deprivation therapy

F/U, follow-up

Patients could not have demonstrable and/or histologically confirmed metastatic or locally recurrent disease demonstrated on bone scan, computed tomography or transrectal ultrasound, or be clinically symptomatic at the time of enrollment. Patients could have received androgen deprivation therapy (ADT), but not within 12 months of entry into the study. In addition, patients had to have a life expectancy of at least one year and performance status of <2 of Zubrod scale. Patients with a known allergic reaction to milk or soy products were excluded.

Twenty men who have had evidence of biochemical relapse after radiation therapy and/or prostatectomy comprised the study cohort. These patients ingested Soy Dream Enriched, Original or Vanilla, soy milk which provided 47 mg of isoflavonoid per 8 oz serving. The patients received three 8 oz servings per day in an open label, nonblinded fashion. No dose escalation or reduction was planned. Compliance was assessed by counting empty soy milk containers and verified by presence of soy components in serum samples.

Pretreatment evaluation included a complete medical history, physical examination (including digital rectal examination), serum PSA, free/total testosterone, lipids, serum isoflavone levels (genistein, daidzein, and equol), and assessment of quality of life (Functional Assessment of Cancer Treatment-Prostate, FACT-P questionnaire). Furthermore, whole blood was obtained prior to initiation of the study to assess for DNA polymorphism. Follow-up serum PSA levels to assess efficacy were obtained at 3, 6, 9, and 12 months after initiation of treatment. Medical history, physical examination, serum testosterone, lipids, isoflavone, and quality of life were assessed at 6 and 12 months after initiation of treatment.

Quality of life prior to and during therapy was measured by the FACT-P questionnaire, which consists of 38 items calculated to evaluate functional impairment and the perceived effect of that impairment on quality of life. The instrument assesses overall and specific aspects of quality of life in five domains (i.e. physical, functional, social, emotional, and prostate related). These include self-reported ability to perform normal physical and social activities, attitude towards self and future, level of physical well-being, and quality of support from friends, family and health care providers. The measure was used to assess the impact of the dietary intervention on quality of life.

At least three pretherapy serum PSAs were available for each subject to compute pretherapy PSA slope. Next, PSA had been measured within days of the time the patient was enrolled in the study. This level was used as study entry level. PSA serum level was measured again 3 months after enrollment in the study, after 6 months, and after 12 months. PSA outcome was evaluated in two ways: a) how response changed over time [i.e. slope of line log (PSA) study entry versus 3 months, versus 6 months, versus 12 months of treatment], and b) as a change on the calculated PSA doubling time before treatment versus PSA doubling time after 3 months, 6 months, and 12 months of treatment. PSA doubling time was calculated using the following formula: PSA doubling time = log 2 × t/[log(final PSA) - log (initial PSA)]. Because pretherapy PSA levels were compared to posttherapy PSA levels, each subject could serve as his own control.

Patients were allowed to continue on therapy until a) they exhibited evidence of PSA progression (as defined by two successive increases in their PSA with an absolute increase of at least 30% above baseline), b) they had clinical evidence or radiographic evidence of distant metastases, or c) patient/physician opted to stop therapy.

### Isoflavone Analysis

A complete LC-MS analysis of patient serum samples for genistein, daidzein and equol (total and aglycone) was performed. The serum samples were both sulfate and glucuronide conjugates which requires hydrolysis with a mixed β-glucuronidase and sulfatase enzyme preparation (*Helix pomatia*; Sigma Chemicals, St Louis, MO) to determine total versus free isoflavone. For each sample, an aliquot was analyzed directly for free isoflavones, while another was enzymatically hydrolysed to determine total isoflavones. Quantitative isoflavone analysis was accomplished using reversed-phase HPLC with UV and mass spectral detection in series [12]. Briefly, samples were prepared for analysis by mixing with ammonium acetate buffer and formic acid prior to multiple extractions in ethyl acetate. Pooled extracts were dried under nitrogen and resuspended in 0.5 ml of 50:50 acetonitrile: 0.2% aqueous formic acid. Samples were then injected onto an Apollo C18 column (Alltech, Deerfield, IL) with Alltech Adsorbosphere HS C18 guard column in a Hewlett-Packard 1100 series liquid chromatograph system (Wilmington, DE). Separation occurred under a linear gradient with mobile phase B (0.1% formic acid in acetonitrile) increasing from 40% to 60% over 60 min. Mobile phase A was 0.1% formic acid in water. The flow rate was 0.40 ml/min with column temperature of 40°C. Free isoflavones and deconjugated glucosides were detected for quantitation using an HP variable wavelength UV detector at 260 nm and quantified against an external standard series. Identification was confirmed using the Finnigan LCQ Ion Trap Mass Spectrometer (Finnigan MAT, San Jose, CA) in positive ion mode with electrospray ionization. Extraction efficiency was determined using the isoflavone biochanin A, with phenolphthalein-glucuronide as deconjugation surrogate.

### Genomic DNA Isolation & Evaluation of AR CAG Polymorphisms

Whole blood was obtained from patients and DNA extracted utilizing QiAamp DNA blood kit (Qiagen, Valencia, CA). Nested PCR reaction was utilized to amplify the CAG polymorphism on the AR gene. Primers for AR gene were designed using Primer Express software (PE-Applied Biosystems). Outside primers for CAG was constructed: 5'-GTGCGCGAAGTGATCCAGAA-3' and 5'-TCTGGGACGCAACCTCTCTC-3' and inside primers 5'-AGAGGCCGCGAGCGCAGCACCTC-3'-fam and 5'-GCTGTGAAGGTTGCTGTTCCTCAT-3'. The inside forward primer was labeled with fluorescent 6-carboxy-fluorescein (FAM). The first PCR reaction consisted of 17 cycles (94°C for 1 min, 55°C for 1 min, and 72°C for 30 sec). The nested PCR used 1 μl of the first PCR product and amplified further for 28 cycles (94°C for 1 min, 66°C for 1 min, and 72°C for 1.5 min). Screening for AR mutations was performed using single-strand conformation polymorphism (SSCP) of PCR amplified AR genomic DNA. The radio-labeled PCR fragment identified above was analyzed by nondenaturing polyacrylamide gel electrophoresis [13]. Fragments showing an aberrant PCR-SSCP pattern on gel were subjected to direct DNA sequencing to document the exact nucleotide base deletion, addition, or change.

### Statistical analysis

A mixed regression model was used to compare the slope of PSA after study entry to that before study entry. The PSA levels for a given patient were treated as repeated measurements and the uniform correlation between PSA measurements was assumed. The slopes before and after study entry were globally compared using all data points in the mixed model. In addition, the slope of PSA after study entry was compared to that before study entry for each individual patient by regular regression analysis. The PSA doubling time before study entry was computed by tlog2/[log(PSA at entry) - log(initial PSA)] and the PSA doubling time after study entry was computed by tlog2/[log(last PSA) - log(PSA at entry)]. Note that the PSA doubling time can be negative if the denominator is negative. The PSA doubling times before and after study entry were compared by the sign test based on paired data. The Wilcoxon signed rank test was performed for other chemistries (Table 2) and QOL. The correlation between AR gene CAG polymorphisms and PSA levels at 12 months after study entry was evaluated by the Spearman's correlation coefficient. All reported p-values were 2-sided. All data were analyzed using SAS version 9.1.3 software.

**Table 2:** Serum PSA, Testosterone, and Cholesterol Before and During Study

**Pt No.**	**PSA at entry (ng/ml)**	**PSA at 12 months (ng/ml)**	**Free PSA at entry (ng/ml)**	**Free PSA at 12 months (ng/ml)**	**Testosterone at entry (ng/ml)**	**Testosterone at 12 months (ng/ml)**	**Free Testosterone at entry (ng/ml)**	**Free Testosterone at 12 months (ng/ml)**	**Cholesterol at entry (mg/dl)**	**Cholesterol at 12 months (mg/dl)**
1	0.57	1.93	0.10	0.381	493	468	N/A	10.1	195	207
2	0.50	0.35	0.08	0.007	331	382	46.7	8.5	148	134
3	1.76	1.62	N/A	N/A	259	263	11.9	N/A	229	N/A
4	1.17	N/A	0.11	N/A	508	N/A	9.3	N/A	109	N/A
5	6.87	11.23	N/A	1.690	324	299	N/A	5.7	203	173
6	5.94	13.41	0.83	2.250	N/A	418	N/A	9.7	140	134
7	1.18	1.73	N/A	0.280	264	118	N/A	5.5	162	141
8	0.67	N/A	0.08	N/A	264	N/A	10.2	N/A	167	N/A
9	0.15	0.12	0.01	N/A	68	68	3.4	N/A	260	197
10	0.4	N/A	N/A	N/A	360	N/A	N/A	N/A	N/A	N/A
11	18.86	N/A	N/A	N/A	137	N/A	4.6	N/A	199	N/A
12	11.21	N/A	1.49	N/A	204	N/A	N/A	N/A	134	N/A
13	0.74	0.67	0.07	N/A	565	N/A	15.6	N/A	319	N/A
14	3.49	3.90	0.22	3.902	280	265	10.4	9.7	151	145
15	1.36	1.59	N/A	N/A	521	487	19.3	N/A	255	235
16	0.50	0.50	N/A	0.020	N/A	281	N/A	12.0	N/A	200
17	2.67	5.15	0.15	0.290	287	382	9.8	0.9	180	170
18	0.59	1.10	N/A	0.100	333	317	4.0	47.6	147	163
19	1.04	N/A	N/A	N/A	N/A	N/A	N/A	N/A	N/A	N/A
20	0.79	1.05	N/A	N/A	299	N/A	10.5	N/A	116	171
Median	1.11	1.61	0.11	0.19	299	308	10.3	9.7	167	171

P value	0.081		0.285		0.064		0.031		0.806	

## Results

### Subjects' characteristics

Twenty men with biochemical recurrent prostate cancer were enrolled. Table 1 shows the demographic, clinical, and pathologic characteristics of the cohort. Compliance was estimated to be high with only two subjects noted not to ingest the required amount of soy milk. Six men did not complete the study: 1 due to side effects, 2 were lost to follow-up, and 3 began ADT. One of the men who had initiation of ADT did so despite no significant increase in serum PSA. The other two patients had a significant increase in serum PSA (PSA doubling time < 3 months). The two non-compliant patients were among the six men who dropped out of the study.

### PSA level – Reduction

Six patients achieved 8%, 9%, 20%, 23%, 30% and 70% reduction in PSA after the treatment, respectively. In addition, one patient had no change in PSA. The remaining 13 patients had increased PSA.

### PSA level – Comparison of Slopes

The slopes of PSA after study entry were compared to that before study entry in a mixed regression model as described in the statistical analysis section. The PSA levels for a given patient were treated as repeated measurements and the uniform correlation between PSA measurements were assumed. Within the mixed regression model, it was estimated that PSA had increased 56% per year before study entry and PSA increased 20% per year for the 12-month study period (*p *= 0.05). There were 6 drop-outs: four had PSA measurements up to 9 months and two had PSA measurements up to 6 months. Since the mixed regression model has the ability to incorporate missing data, the six drop-outs were included in the analysis.

**Figure 1:**
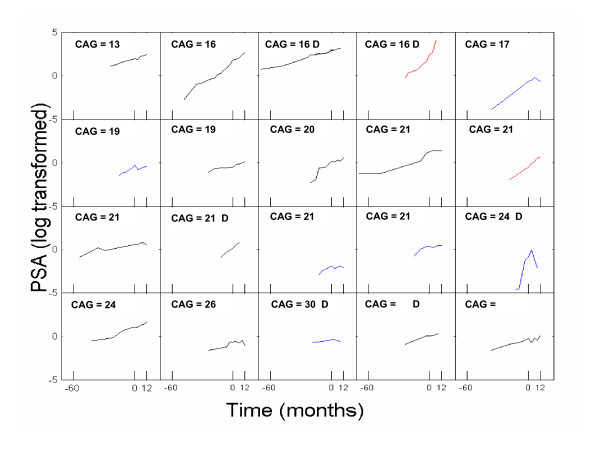
**PSA levels in logarithm scale for each patient.** Blue line (N = 6) represents the slope of PSA during isoflavone therapy was significantly lower than that before the therapy, red line (N = 2) represents the slope of PSA during isoflavone therapy was significantly higher than that before the therapy and black line (N = 12) represents the slope of PSA during isoflavone therapy was unchanged than that before the therapy. CAG, CAG androgen receptor polymorphisms. D, subject who dropped out before completion of the therapy.

The slope of PSA after study entry was also compared to that before study entry for each individual patient. It was found that the slope of PSA after study entry was significantly lower than that before study entry in 6 patients (*p *< 0.05) and the slope of PSA after study entry was significantly higher than that before study entry in 2 patients (*p *< 0.05). For the remaining 12 patients, the change in slope was insignificant (Figure 1). In two drop-outs, the PSA slopes after study entry were significantly higher than that before study entry; in other two drop-outs, the PSA slopes after study entry were significantly lower than that before study entry; in the remaining two drop-outs, the differences in PSA slope were not statistically significant. Drop-outs are noted by "D" on Figure 1.

### PSA doubling time

Changes in PSA after study entry were compared to that before study entry in terms of doubling time. The PSA doubling times before and after study entry were computed for each patient. PSA doubling times were positive in all patients (median = 15 months) before study entry, i.e. PSA levels at study entry were higher than the initial PSA levels. In contrast, the PSA doubling time was negative during the therapy in 6 patients. Note that the doubling time was negative if the last PSA level was lower than the PSA level at study entry. In addition, one patient had no change in PSA and the doubling time was undefined. The PSA doubling time was positive during the therapy for the remaining 13 patients (median = 15 months). Among the 13 patients, 7 had PSA doubling times longer than that before study entry. Improvements have been seen in 14 patients in terms of PSA doubling time (*p *= 0.044, sign test).

### Other chemistries

Free testosterone decreased while on therapy (median 10.3 vs. 9.7 ng/ml, *p *= 0.031). However, neither total testosterone nor cholesterol level was significantly manipulated during isoflavone therapy (Table 2).

### Isoflavone Level and AR Molecular Weight

Median total genistein, daidzein, and equol prior to the start of therapy were <0.002, 0.012, and 0.023 μg/ml, respectively. Serum isoflavone levels measured at 6 months are depicted in Table 3. Among 16 men with available data, 12 men (75%) demonstrated significant (> 2 × CTL, CTL = 0.023 μg/ml) levels of total equol and 10 of 16 (63%) had significant (> 2 × CTL, CTL = 0.009 μg/ml) levels of free equol present in serum. In the study cohort, median genistein level was 0.524 μg/ml (range < 0.002 to 2.071), median daidzein levels was 0.468 μg/ml (range 0.005 to 0.817), and median equol level was 0.115 μg/ml (range <0.001 to 0.523). Median free genistein level was 0.012 μg/ml (range <0.002 to 0.019), median free daidzein level 0.016 μg/ml (range 0.003 to0.434), and median free equol level was 0.040 μg/ml (range <0.001 to 1.328). The correlations of free equol and total equol with PSA level at 12 months were 0.11 and 0.39, respectively (p-values = 0.71 and 0.19). Correlations of genistein (free and total) and daidzen (free and total) with PSA level at 12 months were not significant either.

**Table 3:** Total and Free Serum Isoflavones and Androgen Receptor CAG repeat

**Patient No.**	**Total Genistein (μg/ml)**	**Total Daidzein (μg/ml)**	**Total Equol (μg/ml)**	**Androgen Receptor CAG repeat**
1	0.709	0.735	0.115	21
2	1.284	0.660	0.063	26
3	0.189	0.468	0.331	21
4	0.555	0.409	0.306	21
5	0.102	0.572	0.523	13
6	0.739	0.468	0.039	16
7	2.071	0.602	0.329	20
8	N/A	N/A	N/A	30
9	0.022	0.014	0.020	21
10	N/A	N/A	N/A	24
11	0.023	0.568	<0.001	16
12	0.186	0.063	0.061	16
13	<0.002	0.005	0.048	19
14	0.058	0.192	0.050	21
15	1.049	0.580	0.188	21
16	0.492	0.170	0.062	17
17	1.989	0.817	0.492	24
18	<0.002	0.021	0.119	19
19	N/A	N/A	N/A	N/A
20	N/A	N/A	N/A	N/A
Median	0.524	0.468	0.115	---

N/A, not assessed (adequate sample unable to be obtained)

μg, microgram

mL, milliliter

MW, molecular weight

**Figure 2:**
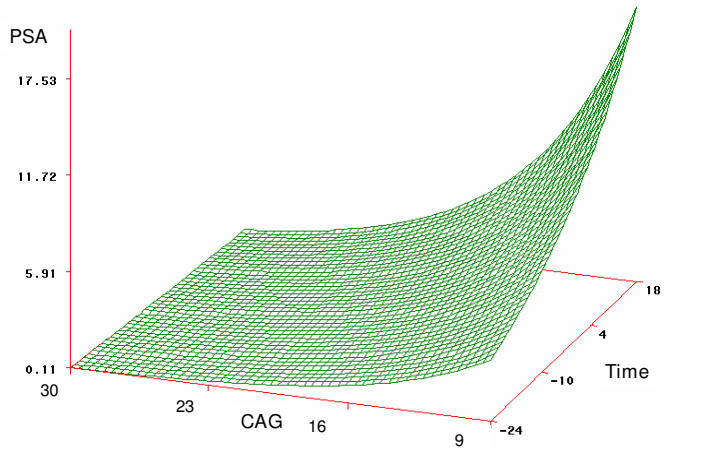
Effect of PSA vs. CAG Polymorphism MW over Time. MW, molecular weight.

There were 9 documented AR gene CAG polymorphisms in our cohort (Table 3). Eight men had ≤ 20 CAG repeat. A longer CAG polymorphism was associated with a lower PSA level at 12 months after study entry (Spearman's correlation coefficient = -0.63, *p *= 0.05) and a slower rise in PSA over time (Figure 2).

### Quality of Life

**Figure 3:**
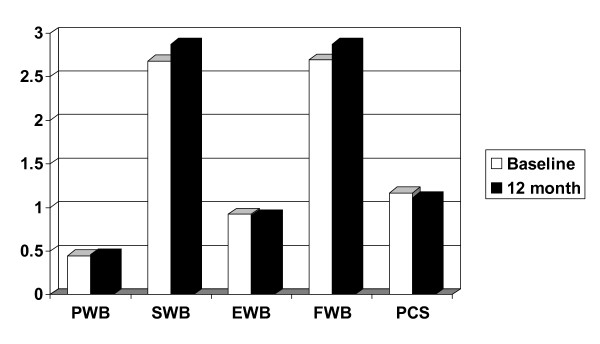
**Baseline (white) and 12 month (black) FACT-P Quality of Life questionnaire in 20 men with biochemical recurrent prostate cancer after previous therapy.** (PWB, physical well being; SWB, social well being; EWB, emotional well being; FWB, functional well being; PCS, prostate cancer specific).

There was no significant difference in the five domains of the FACT-P between pretherapy and on therapy (Figure 3). Side effects were minimal. One patient of 20 (5%) reported diarrhea and withdrew from the study. No other side effects were reported.

## Discussion

Numerous phase I and II clinical trials have studied men with biochemical recurrence (PSA only) prostate cancer after previous therapy. To date no randomized clinical trial has demonstrated a survival advantage of salvage therapies in this cohort. Thus, these men pose a difficult therapeutic dilemma. The majority of them are asymptomatic. Therapeutic maneuvers with hormonal ablation, chemotherapy, and experimental agents may have significant side effects. It is a difficult to persuade an asymptomatic man to begin a potential toxic regimen with unknown benefit. Because of these reasons, men with PSA only recurrence are excellent candidates for effective holistic regimens. The goal of such treatment is to delay or prevent disease progression and thereby prolong the time until ADT. Furthermore, slowing the rate of PSA rise may have an effect on overall survival.

We treated 20 men with 141 mg of isoflavone per day for 12 months. Our dose regimen was based on 1) 22 randomized studies performed to assess cardiovascular disease[14] and 2) ease of administration (1–8 oz glass of soy milk for breakfast, lunch, and dinner). Because the only sign of recurrent disease after failed therapy was rising serum PSA profile, PSA response was the primary end point measure of our study. In general, serum PSA levels at study entry were not significantly different after 12 months of therapy (mean 3.02 vs. mean 3.17, respectively). Of the 20 patients, the slope of PSA after study entry was significantly lower than that before study entry in 6 patients, significantly higher than that before study entry in 2 patients, and unchanged in the remaining 12 patients. Thus, we were able to demonstrate a decline in the rise of serum PSA after the initiation of soy milk, thus demonstrating isoflavones biologic activity in prostate cancer patients. Furthermore responders started with a lower serum PSA level.

Several novel phase II non-randomized clinical trials have been reported in patients with biochemical recurrent prostate cancer. 1,25-dihydroxyvitamin D3 can result in differentiated cancer cells and induce apoptosis [15]. Gross and others demonstrated that 0.5 μg-2.5 μg of 1,25-dihydroxyvitamin D3 (Calitriol) daily slowed the rate of serum PSA rise in 6 of 7 patients. Unfortunately, hypercalciuria developed in all patients, with one developing a renal calculus and another developing renal insufficiency [16]. It is possible that the precursor to 1,25-dihydroxyvitamin D3, 25 hydroxyvitamin D3 may be administered with significantly less side effects. Research suggests that the prostate, similar to the kidney, possesses the enzyme to convert 25-hydroxyvitamin D3 to 1,25-dihydroxyvitamin D3, thus enabling it to exert their effect [17]. Next, Woo reported 9 out of 15 patients with PSA relapse prostate cancer after definitive therapy had stable or decreasing serum PSA levels when treated with 2,000 IU (50 μg) of 25-dihydroxyvitamin D3 (cholecalciferol) daily. No adverse side effects were reported [18]. Pruthi and others reported the effects of celecoxib (COX-2 inhibitor) 400 mg given orally twice daily in 40 men with PSA recurrent prostate cancer after definitive treatment. Eleven men had a decline in serum PSA and 8 had a stabilization of PSA. One patient was removed from the study for a presumed transient ischemic attack [19]. Researchers from Wake Forest reported that 15–120 mg/day of lycopene supplementation was safe and well tolerated in a cohort of 36 men with PSA recurrent prostate cancer. However, no serum PSA responses were observed and 37% of patients had PSA progression [20].

Hussain and others reported on a heterogeneous group of men with prostate cancer who were treated with 100 mg of soy isoflavone (Novasoy) twice daily for a maximum of 6 months. There was a decrease in the rate of the rise of serum PSA in men with PSA recurrent disease. The rates of rise decreased from 14% to 6% while on study [21]. These results are consistent with our study, where the rates of rise decreased from 56% to 20% while on study. Our more favorable results may be due to longer treatment duration (6 months vs. 12 months). Taken together, the data from the two clinical trials in men with PSA recurrent prostate cancer treated with isoflavone are encouraging and should be further studied. Ideally these studies should be combined with genomics or proteomics assays to 1) determine the profile of treatment responders, and 2) attempt to elucidate mechanistic pathways that can be exploited by combining these agents with other holistic drugs or with a more conventional therapy.

Isoflavones in the soy milk included genistein and daidzein. Numerous studies have demonstrated the cytostatic and cytotoxic effect of genistein in various malignancies including breast, lung, melanoma, prostate, head and neck squamous cell carcinoma, leukemia, and lymphoma [22]. Genistein has a heterocyclic diphenolic structure similar to estrogen [22]. Because of the similar structure, it is proposed that soy (genistein) exerts its effect on cells by interacting with hormone receptors. The exact mechanism of genistein's anti-tumoral effects is unknown. Proposed mechanisms are perturbations in cell cycle, decreased proliferation, decreased angiogenesis, and increased apoptosis. In addition to genistein and daidzein, another metabolite of isoflavone is equol. Equol is produced via the bacterial conversion of the soy isoflavone daidzein in the intestines [23]. It is estimated that approximately 30% of Americans can convert diadzein to equol, however the real percentage is unknown seeing that no large study has assessed this in the general American population. The percentages are higher (> 80%) in Chinese and Japanese. Similar to the other isoflavone metabolite, equol has a chemical structure related to estrogen and is known to interact with estrogen receptors [23]. Over two-thirds of the men in our study were able to convert daidzein to equol. Overall, median total equol level was 0.115 μg/ml (range <0.001 to 0.523) and median free equol level was 0.040 μg/ml (range <0.001 to 1.328). There could be genetic differences in isoflavone metabolism in each racial group, however due to the small numbers this could not be assessed.

The CAG repeat polymorphisms of the androgen receptor gene have been associated with an increased prostate cancer risk and the repeat length has been correlated with cancer stage and grade at presentation [24]. Investigators have also correlated CAG repeat polymorphism of the AR gene to response to ADT [25]. Similarly, in this small study, we demonstrated that subjects with CAG polymorphisms molecular weight > 22 were more likely to respond to the experimental therapy (Figure 2). In future studies, CAG polymorphisms may be used to determine eligibility into these trials (i.e., subjects with CAG polymorphisms ≥ 22 will be enrolled). Due to the small numbers in the current study, we could not correlate CAG polymorphisms with equol production which could be a fascinating correlation.

There are several limitations to this study. First, this is a small study that was terminated early due to poor accrual. The planned power of 60% was not attained. It is possible that with a larger number of subjects, a more significant change in serum PSA while on therapy could have been demonstrated. Furthermore there was a substantial drop out rate in this study. Previous studies have commented on the difficulty of performing clinical trials in an inner-city population such as ours [26,27]. In addition, this phase II study did not have a control group and the therapy was neither randomized nor blinded. Though not ideal, this study design which was employed by other groups [16,18-20] would provide useful data on the feasibility and utility of isoflavone in this cohort prior to embarking on a larger, costly phase II or even phase III study. Last, serum PSA was the primary endpoint of this study. We acknowledge the limitations of serum PSA in this study (i.e., fluctuations). But to date there are no other prognostic markers for prostate cancer in this cohort. To decrease the chance of detecting 'noise' (or common fluctuations of serum PSA levels), we assessed not absolute PSA but PSA trends. Nevertheless, the results of this study suggest biologic activity with the isoflavone diet in men with biochemical recurrent prostate cancer

## Conclusion

Our findings show that isoflavone was a well-tolerated alternative to expectant management or early ADT in men with biochemical recurrent prostate cancer. Isoflavone may slow the rate of increase of serum PSA in a subset of patients with biochemical progression after radiation therapy or radical prostatectomy. The findings reported herein warrant further investigation in a larger cohort of patients. In these larger studies, correlative biomarkers should be explored to assist with elucidating a possible mechanism of action with isoflavones in prostate cancer.

## Competing interests

The authors declare that they have no competing interests.

## Authors' contributions

JMP was study coordinator, WWT physician of 9 patients who assisted with recruitment and follow-up, SA performed CAG portion of the project, MC and WH were statisticians, KTS assisted with interpretation of isoflavone serum levels, CJR was study PI.

## Pre-publication history

The pre-publication history for this paper can be accessed here:


